# Prognostic value of growth differentiation factor-15 in patients with coronary artery disease: A meta-analysis and systematic review

**DOI:** 10.3389/fcvm.2023.1054187

**Published:** 2023-02-10

**Authors:** Song Zhang, Panpan Hao, Jiaxin Li, Qi Zhang, Xiaoying Yin, Jiali Wang, Yuguo Chen

**Affiliations:** ^1^Department of Emergency and Chest Pain Center, Qilu Hospital of Shandong University, Jinan, China; ^2^Shandong Provincial Clinical Research Center for Emergency and Critical Care Medicine of Shandong Province, Institute of Emergency and Critical Care Medicine of Shandong University, Qilu Hospital of Shandong University, Jinan, China; ^3^The Key Laboratory of Cardiovascular Remodeling and Function Research, Chinese Ministry of Education, Chinese Ministry of Health and Chinese Academy of Medical Sciences, Jinan, China; ^4^The State and Shandong Province Joint Key Laboratory of Translational Cardiovascular Medicine, Qilu Hospital of Shandong University, Jinan, China

**Keywords:** growth differentiation factor-15, coronary artery disease, myocardial infarction, independent prognostic value, systematic review and meta-analysis

## Abstract

**Background and aims:**

The predictive value of growth differentiation factor-15 (GDF-15) for individual cardiovascular outcomes remained controversial in patients with coronary artery disease (CAD). We aimed to investigate the effects of GDF-15 on all-cause death, cardiovascular death, MI and stroke in CAD patients.

**Methods:**

We searched PubMed, EMBASE, Cochrane library and Web of Science till 30 December, 2020. Hazard ratios (HRs) were combined with fixed or random effect meta-analyses. Subgroup analyses were performed in different disease types. Sensitivity analyses were used to evaluate the stability of the results. Publication bias was tested using funnel plots.

**Results:**

A total of 10 studies with 49,443 patients were included in this meta-analysis. Patients with the highest GDF-15 concentrations had significantly increased risk of all-cause death (HR 2.24; 95% CI: 1.95–2.57), cardiovascular death (HR 2.00; 95% CI: 1.66–2.42), MI (HR 1.42; 95% CI: 1.21–1.66) after adjusting clinical characteristics and prognostic biomarkers (hs-TnT, cystatin C, hs-CRP, and NT-proBNP) but except for stroke (HR 1.43; 95% CI: 1.01–2.03, *p* = 0.05). For the outcome of all-cause death and cardiovascular death, subgroup analyses revealed consistent results. Sensitivity analyses showed that the results were stable. Funnel plots showed that there was no publication bias.

**Conclusion:**

In CAD patients with elevated GDF-15 levels on admission, there were independently significant risks for all-cause death and cardiovascular death. The highest concentrations of GDF-15 had a lower predictive effect on MI than all-cause death and cardiovascular death. The association of GDF-15 with the outcome of stroke needs to be further studied.

## 1. Introduction

Coronary artery disease (CAD) is the most common cause of death and disability around the world, and accounts for approximately 30% of all deaths (1–3). Over 23 million human beings are anticipated to suffer from cardiovascular disease before 2030 and CAD is the most common disease type of it (4). It is very important to recognize patients who are at high risks for future adverse cardiovascular events. Traditional biomarkers have a vital function of assisting in predicting future cardiovascular risks (5), such as NT-proBNP and hs-cTnT (6). The independent prognosis effect of novel biomarkers in CAD needs to be determined by more results.

Growth differentiation factor-15 (GDF-15) reflects cardiovascular function and disease status, which is an inflammation-related biomarker and belongs to the transforming growth factor-β (TGF-β) cytokine superfamily. While weakly expressed in physiological conditions, GDF-15 is strongly induced under pathological stress response related to inflammation or tissue injury (7). The elevated GDF-15 levels had been detected in human macrophages of the atherosclerotic plaque (8). A serious of clinical research has been explored the association between GDF-15 concentrations and the prognostic effect of cardiovascular diseases. However, after adjusting for clinical characteristics and biomarkers such as hs-troponin T, cystatin C, high-sensitivity C-reactive protein (hs-CRP), N-Terminal B-Type natriuretic peptide (NT-proBNP), the independent predictive effect of GDF-15 on all-cause death, cardiovascular death, MI and stroke did not yield consistent results in CAD patients. Several studies have shown a diminished association of GDF-15 with MI after adjustment for clinical features and other prognostic biomarkers (9–12).

We aimed to focus on the independent predictive effect of GDF-15 on individual cardiovascular events in CAD patients in this meta-analysis.

## 2. Methods

### 2.1. Data sources and study selection

This meta-analysis and systematic review was conducted according to the guidelines of Preferred Reporting Items for Systematic Reviews and Meta-Analyses (PRISMA) (13). We used the following retrieval strategies to screen studies with the terminology in the database of PubMed, Embase, Web Of Science and Cochrane: (“coronary artery disease” or “coronary heart disease” or “ischemic heart disease” or “coronary atherothrombotic heart disease”) and (“growth differentiation factor 15” or “macrophage inhibitory cytokine 1” or “prostate differentiation factor” or “differentiation factor, prostate” or “GDF-15”) and (“prognosis” or “diagnosed” or “cohort” or “cohort studies” or “predictor” or “death” or “models, statistical”). The inclusion criteria of potential studies were: (1) patients were diagnosed with CAD including UA, NSTEMI, STEMI, and stable CAD, (2) all studies were written in English and the inclusion and exclusion criteria of each study were clearly defined, (3) studies had a specific interpretation and assessments of the outcomes, as well as a sufficient follow-up period, and (4) studies provided enough data such as hazard ratio (HR) and 95% confidence intervals (CIs) according to the circulating levels of GDF-15.

### 2.2. Data extraction

Two investigators (SZ, JW) independently screened all studies and extracted the data using the data collection forms. Disagreements were settled by a third investigator (PH). We recorded the study characteristics, including publication year, the first author, number of patients, research design, the type of disease, follow-up duration, classification of GDF-15 concentrations (tertile or quartile). We recorded individual adverse cardiovascular events such as all-cause death, cardiovascular death, recurrent MI as well as stroke.

### 2.3. Statistical analysis

We calculated the available data from included studies with the data statistical software of Review Manager 5.3 (RevMan 5.3). We used direct extraction or indirect calculation methods to extract data from the original literatures and then analyzed the rate of individual cardiovascular events. We used fixed (Inverse Variance) or random effects methods to estimate summary HRs and 95% CIs for the outcomes. The heterogeneity of the included studies was assessed by *I*^2^ values which *I*^2^ > 50% and *p* < 0.05 were defined as the presence of significant heterogeneity. Data are presented as summary HRs with 95% CI and two-tailed *p* values. *p* < 0.05 was considered statistically significant.

The extraction of HR values and 95% CI corresponded to the lowest tertile or quartile group. Make adjustments using the following model, Model 1: Clinical characteristics included age, sex, previous MI, previous percutaneous coronary intervention (PCI), previous coronary artery bypass grafting (CABG), body mass index (BMI), diabetes mellitus, hypertension, history of heart failure, smoking. Model 2: traditional biomarkers such as hs-troponin T, NT-proBNP, cystatin C, and hs-CRP in addition to Model 1.

The highest levels of GDF-15 were defined as the fourth quartile in the quartile classification and the third quartile (>1,800 ng/L) in the tertile classification. The third quartile in the quartile classification was defined as the middle level of GDF-15, which was merged with the 1,200–1,800 ng/L levels of GDF-15. Subgroup analyses were performed to explore the differences across the disease types of CAD (ACS or stable CAD). Sensitivity analysis was performed by removing individual studies that did not classify as the <1,200 ng/L, 1,200–1,800 ng/L, >1,800 ng/L, and distinguish clinical trials or cohort studies, the purpose of it was to evaluate the robustness of the outcomes in different aspects, because the included patients and interventions are often different. We used forest plots to present the results with the combination of graphic and data. And publication bias was evaluated by funnel plots.

### 2.4. Quality assessment

Newcastle-Ottawa Scale (NOS) score was used for evaluating the quality of the literatures by two investigators (14). The NOS included three main aspects: selection of the study population, comparability of the study groups, and assessment of the study results. The highest score on this scale is 9, if the score of the study is higher, it is considered to have high methodological quality. Discrepancies in the data were adjudicated by a third investigator. The included studies had a score equal to or greater than five in NOS (Supplementary Table 1).

## 3. Results

### 3.1. Study selection

**Figure 1:**
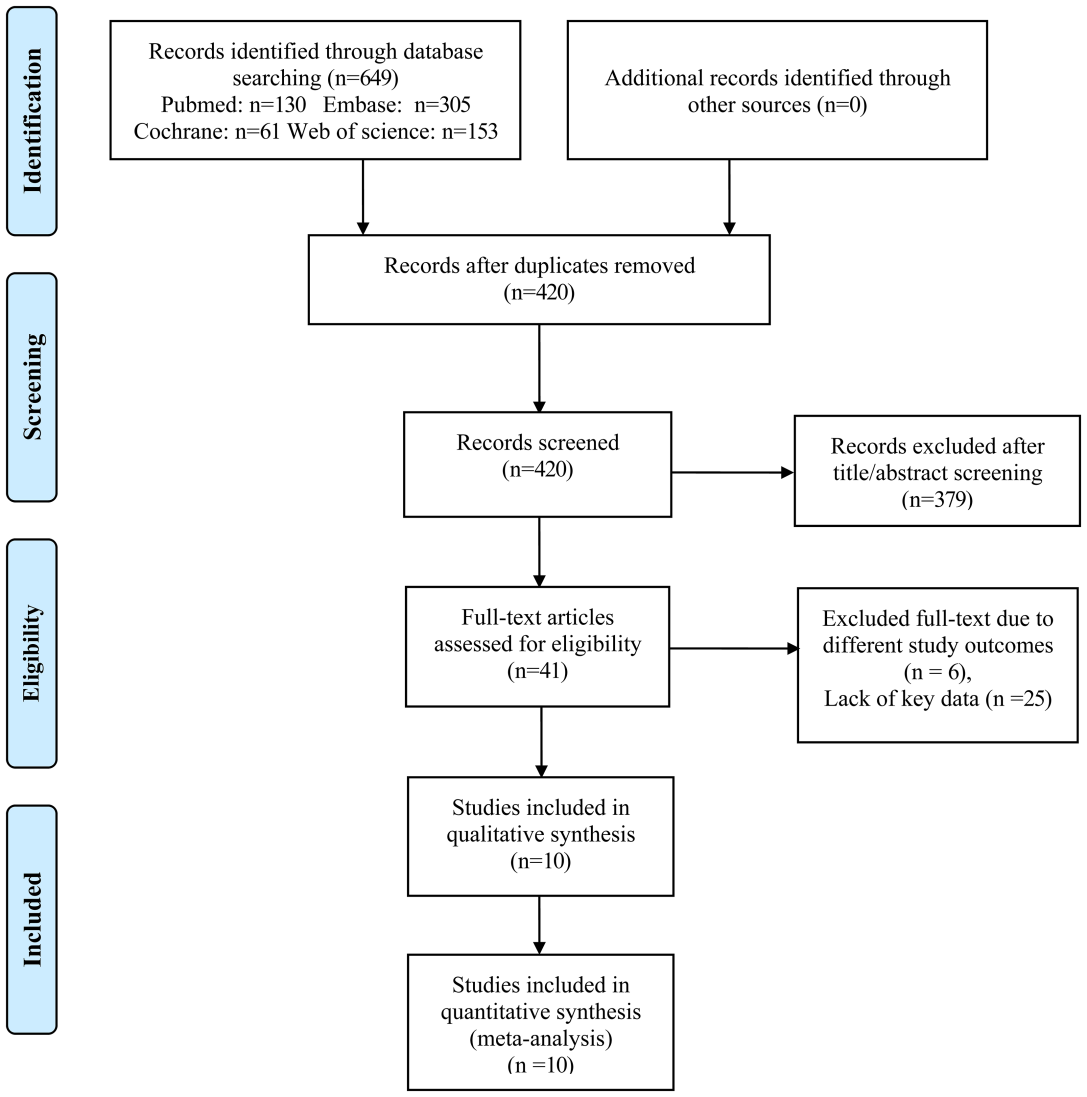
Flowchart showing selection of studies.

A total of 649 articles (130 articles from PubMed, 305 articles from EMBASE, 61 articles from Cochrane, and 153 articles from Web of Science) were identified initially. Furthermore, we screened 420 articles after removing 229 duplicate articles. 379 articles were excluded according to the titles and abstracts. We further evaluated 41 articles by reading the full text. Among them, six articles were excluded due to different outcomes and 25 articles were excluded for lack of key data. Finally, 10 articles matched our meta-analysis condition (10–12, 15–21), the complete process of selection of studies following PRISMA guidelines for inclusion is summarized in Figure 1. The other 31 studies were excluded due to insufficient data such as HR and 95% CIs corresponding to the middle and the highest concentrations of GDF-15 after adjustment for clinical characteristics and other biomarkers or non-conformity of research content.

### 3.2. Study characteristics

Ten studies involving 49,443 patients in this meta-analysis in the aggregate and the median follow-up time was 3.89 years. Seven articles included only patients with ACS, two studies contained only stable CAD patients, one article included both ACS and stable CAD patients, and one article did not provide the types of CAD. The classification of GDF-15 concentrations in six studies was carried out according to the accepted cut-off values (<1,200 ng/L, 1,200–1,800 ng/L, >1,800 ng/L) (22) and other four studies used GDF-15 quartile groups. The study type of the four studies were designed as cohort studies and other six studies were clinical trials. The clinical characteristics of the 10 articles are shown in Table 1.

**Table 1:** Characteristics of studies included in the meta-analysis.

Author	Year	Sample size	Study design	Patients	Nationality	Male(%)	Age (years)	Follow-up (years)	Outcomes	GDF-15 (ng/L)
Peiró ÓM, et al.	2019	358	Cohort	ACS	Spain	72.6	64.8	6.5	All-cause death	<1,200, 1,200–1,800, >1,800
Held C, et al.	2017	14,577	trial	Stable CAD	Sweden	81.5	65.3	3.7	All-cause death, CV death, MI, stroke	<915, 915–1,253, 1,253–1,827, >1,827
James SK, et al.	2016	16,876	Trial	ACS	Sweden	71.3	62.0	1.0	All-cause death, CV death, MI, stroke	<1,145, 1,145–1,550, 1,550–2,219, >2,219
Schopfer DW, et al.	2014	984	Cohort	Stable CAD	America	81.5	66.7	8.9	All-cause death, CV death, MI, stroke	<1,770, 1,770–2,660, >2,660
Damman P, et al.	2014	1,151	Trial	NSTEMI	Netherlands	73.1	62.2	5.0	All-cause death, CV death, MI	<1,200, 1,200–1,800, >1,800
Kempf T, et al.	2009	2,229	Cohort	CAD	Germany	77.7	61.6	3.6	CV death	<1,200, 1,200–1,800, >1,800
Velders MA, et al.	2015	5,385	Trial	STEMI	America	77.4	59.0	0.8	CV death, MI	<1,116, 1,116–1,492, 1,492–2,120, >2,120
Li M, et al.	2020	3,641	Cohort	CAD	China	72.3	61.4	6.4	All-cause death	<1,200, 1,200–1,800, >1,800
Kempf T, et al.	2007	741	Trial	STEMI	America and Europe	70.2	67.0	1.0	All-cause death	<1,200, 1,200–1,800, >1,800
Bonaca MP, et al.	2011	3,501	Trial	ACS	America and Europe	78.9	58.1	2.0	All-cause death, MI	<1,200, 1,200–1,800, >1,800

ACS, acute coronary syndrome; CAD, coronary artery disease; CV death, cardiovascular death; GDF-15, growth differentiation factor-15; MI, myocardial infarction; NSTEMI, non-st-segment elevation myocardial infarction; STEMI, st-segment elevation myocardial infarction.

### 3.3. The association of elevated GDF-15 levels with individual outcomes in CAD patients

#### 3.3.1. All-cause death

**Figure 2:**
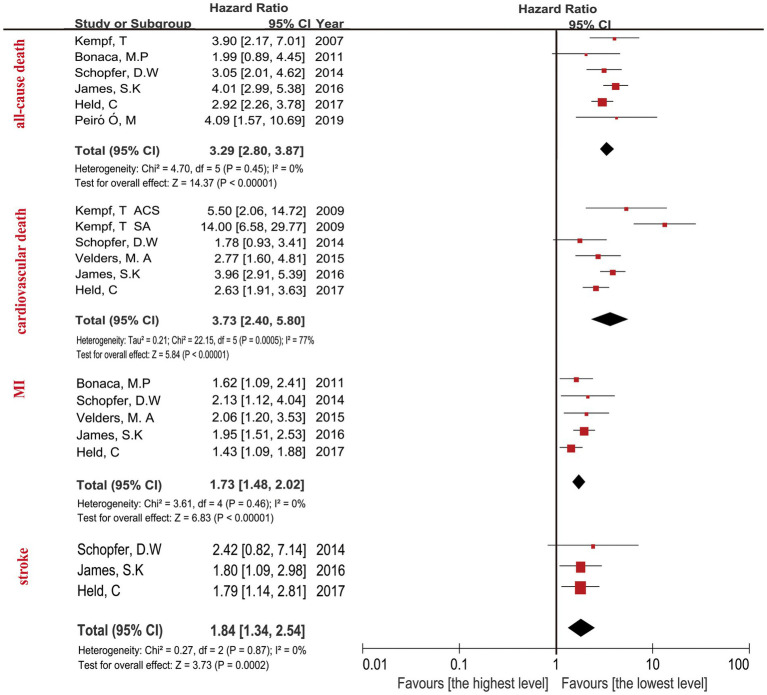
Forest plot showing the HR and 95% CI of individual cardiovascular events for studies comparing the highest and lowest concentrations of GDF-15 after adjustment clinical characteristics.

**Figure 3:**
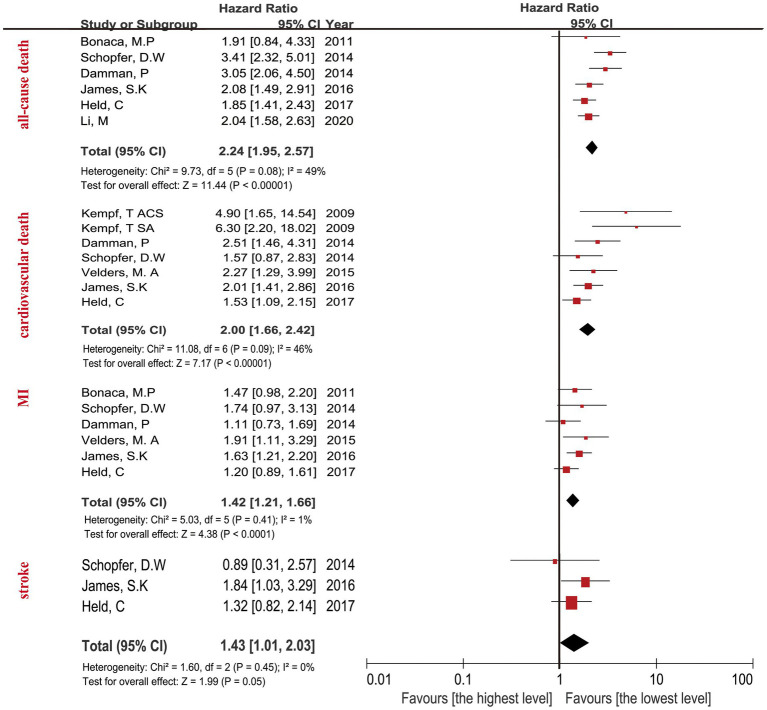
Forest plot showing the HR and 95% CI of individual cardiovascular events for studies comparing the highest and lowest concentrations of GDF-15 after adjustment clinical characteristics and other biomarkers.

The highest concentration of GDF-15 revealed a HR of 3.29 (95% CI: 2.80–3.87; *p* < 0.0001) when compared with the lowest GDF-15 after adjusting clinical characteristics (Figure 2) and a HR of 2.24 (95% CI: 1.95–2.57; *p* < 0.0001) after adjusting clinical characteristics and biomarkers (Figure 3). The predictive effect of GDF-15 at middle concentration was shown in Supplementary Table 2, which indicated a lower risk of all-cause death (HR 1.38; *p* = 0.001; 95% CI: 1.13–1.67) after adjustment for clinical characteristics and other biomarkers.

#### 3.3.2. Cardiovascular death

The relationship between the highest GDF-15 values and cardiovascular death in CAD patients revealed a HR of 3.73 (95% CI: 2.40–5.80; *p* < 0.0001) after adjusting clinical characteristics in Figure 2, and a HR of 2.00 (95% CI: 1.66–2.42; *p* < 0.0001) after adjusting clinical characteristics and biomarkers (Figure 3). The predictive effect of GDF-15 at middle concentration revealed a HR of 1.40 (95% CI: 1.06–1.86; *p* = 0.02) with the addition of adjustment for clinical characteristics and traditional biomarkers in Supplementary Table 2, which indicated the risk of middle levels of GDF-15 was lower than the highest GDF-15 levels.

#### 3.3.3. MI

The MI in CAD patients with the highest levels of GDF-15 revealed a HR of 1.73 (95% CI: 1.48–2.02; *p* < 0.0001) when compared with the lowest GDF-15 after adjusting clinical characteristics in Figure 2, and a HR of 1.42 (95% CI: 1.21–1.66; *p* < 0.0001) after adjusting clinical characteristics and biomarkers in Figure 3. The MI in CAD patients with the middle concentrations of GDF-15 revealed a HR of 1.23 (95% CI: 0.96–1.58; *p* = 0.11) when compared with the lowest GDF-15 after adjustment for clinical characteristics and other biomarkers in Supplementary Table 2, which indicated that no significant association of middle concentrations of GDF-15 was found with MI. The 95% CI of MI was on the far left and there was no significant overlap when compared with the 95% CI of all-cause death and cardiovascular death according to Figures 2, 3, which confirmed that the highest concentrations of GDF-15 had a lower predictive effect on MI than all-cause death and cardiovascular death in patients with CAD.

#### 3.3.4. Stroke

For the stroke analyses, three studies had the data about the highest GDF-15 concentrations compared with the lowest GDF-15 levels after adjustment for clinical characteristics, indicating the combined HR of 1.84 (95% CI: 1.34–2.54; *p* = 0.0002) in Figure 2, the combined HR value was 1.43 (95% CI: 1.01–2.03; *p* = 0.05) with the addition of other biomarkers in Figure 3. The predictive effect of GDF-15 at middle concentration is shown in Supplementary Table 2, which increased the risk of stroke (HR 1.65; 95% CI: 1.18–2.31, *p* = 0.004) after adjustment for clinical characteristics and other biomarkers.

### 3.4. The effects of GDF-15 on all-cause death, cardiovascular death and MI in acute coronary syndrome and stable angina pectoris patients

The highest GDF-15 concentrations increased the risk of all-cause death (HR 2.39; 95% CI: 1.88–3.05 *p* < 0.0001; Supplementary Figure 2), cardiovascular death (HR 2.27; 95% CI: 1.76–2.93, *p* < 0.0001; Supplementary Figure 3), MI (HR 1.49; 95% CI: 1.23–1.82, *p* < 0.0001; Supplementary Figure 4) in patients with ACS after adjustment for clinical characteristics and the prognostic biomarkers. The predictive effect was similar in patients with stable CAD, revealed the HR of 2.47 (95% CI: 1.36–4.50, *p* = 0.003; Supplementary Figure 2) in the outcome of all-cause death, the similar association was sustained in cardiovascular death (HR 2.08; 95% CI: 1.11–3.89, *p* = 0.02; Supplementary Figure 3) and MI (HR 1.29; 95% CI: 0.99–1.68, *p* = 0.06; Supplementary Figure 4). The predictive effect of the highest concentrations of GDF-15 on all-cause death and cardiovascular death persisted in subgroup of both ACS and stable CAD. But the highest concentrations of GDF-15 cannot predict MI in stable CAD patients.

### 3.5. The predictive effect of GDF-15 on all-cause death, cardiovascular death and MI at different follow-up times

At long period of follow-up time (>1 year), the highest levels of GDF-15 increased the risk of all-cause death (HR 2.37; 95% CI: 1.84–3.06, *p* < 0.0001; Supplementary Figure 5), cardiovascular death (HR 2.38; 95% CI: 1.49–3.79, *p* = 0.0003; Supplementary Figure 6), MI (HR 1.29; 95% CI: 1.06–1.57, *p* = 0.01; Supplementary Figure 7) when compared with the lowest levels after adjusting clinical features and biomarkers. And these risks were sustained in all-cause death (HR 2.08; 95% CI: 1.49–2.91; Supplementary Figure 5), cardiovascular death (HR 2.08; 95% CI: 1.54–2.81, *p* < 0.0001; Supplementary Figure 6), MI (HR 1.69; 95% CI: 1.30–2.20, *p* < 0.0001; Supplementary Figure 7) with the follow-up time ≤1 year. The results indicated that the predictive effect of the highest concentrations of GDF-15 on all-cause death and cardiovascular death persisted regardless of follow-up time. The predictive effect of the highest levels of GDF-15 on MI was mild especially in the long-term follow-up.

### 3.6. Sensitivity analyses

The sensitivity analyses showed the results were robust with no significant change after removing a few studies. The highest levels of GDF-15 independently increased the risk of all-cause death (HR 2.27; 95% CI: 1.85–2.80), cardiovascular death (HR 3.29; 95% CI: 2.12–5.11), MI (HR 1.48; 95% CI: 1.23–1.78) by removing a small number of studies which GDF-15 concentrations were not classified at the same method. The results were similar after removing cohort studies.

### 3.7. Publication bias

The funnel plot for individual outcomes showed that no publication bias was observed in Supplementary Figure 1.

## 4. Discussion

In CAD patients with the highest concentrations of GDF-15 on the baseline, there was a significant risk of all-cause death, cardiovascular death besides the influence of clinical characteristics and hs-troponin T, cystatin C, hs-CRP, NT-proBNP in this meta-analysis. This relationship was also existed in different disease types as well as different follow-up time, which indicates that the admission detection results of GDF-15 can provide information on the prognosis of all-cause death, cardiovascular death in CAD patients.

The highest concentrations of GDF-15 has a poor predictive value for MI in patients with CAD, especially for ACS, and these effect was weak than all-cause death and cardiovascular death, but this prognosis value was not existed in stable CAD patients. Middle concentrations of GDF-15 had no predictive effect on MI in patients with CAD. With the further increase of GDF-15 concentrations, the difference between the highest concentrations of GDF-15 and the lowest levels of GDF-15 was more obvious in the outcome of MI. However, the predictive effect of GDF-15 on stroke was unclear. More studies are needed to explore the relationship between GDF-15 levels and the outcome of stroke.

### 4.1. The elevated GDF-15 independently predicts cardiovascular events in addition to familiar biomarkers

Previous studies have indicated that GDF-15 concentrations are associated with age, diabetes mellitus, current smoking status, hs-CRP, NT-proBNP, and renal dysfunction independently in CAD patients (23). In patients with previous MI or heart failure, the GDF-15 concentrations are higher than those without these medical history (9, 16, 17, 20, 21, 24, 25). Previous meta-analyses did not study the independent predictive effect of GDF-15 after adjustment for other biomarkers such as hs-troponin T, cystatin C, hs-CRP, NT-proBNP. The measurement of plasma GDF-15 concentrations can be informative in addition to providing clinical features and established cardiovascular risk factors.

We found that the relationship between the highest concentrations of GDF-15 and all-cause death, cardiovascular death persisted after adjusting clinical characteristics and other biomarkers, suggesting that GDF-15 and other biomarkers reflect nonoverlapping disease pathways. A serious of previous research confirmed that GDF-15 levels were related to biomarkers indicative myocardial injury and dysfunction (troponins, NTpro-BNP), renal dysfunction (cystatin C), and inflammatory activity (hs-CRP) (9, 20, 21, 24, 26–28), our meta-analysis confirmed the independent predictive effects of high concentrations of GDF-15 on all-cause death, cardiovascular death and MI in patients with CAD, which indicated the highest concentrations of GDF-15 measured on admission exerted a direct effect on the progression of CAD. This could provide insight into the understanding of GDF-15.

GDF-15 demonstrates the different aspects of development, progression and prognosis in coronary artery disease which are not implicated by other risk predictors and biomarkers. The available data can confirm the degree to which GDF-15 testing adds to the prognostic information conveyed by troponin, cystatin C, hs-CRP, and NT-proBNP and other clinical factors (such as diabetes, hypertension, age, and gender) to a certain extent. Although the studies we included show that GDF-15 provides independent prognostic information, further clinical researches are needed to establish the value of this association in clinical decision making. We suggest that any risk assessment cannot rely solely on the GDF-15 level. The studies included in our meta-analysis were association analyses to explore the relationship between GDF-15 and individual cardiovascular events. A more deterministic analysis is needed to verify this result.

### 4.2. The prognostic effect of the highest concentrations of GDF-15 on death was similar in ACS and stable CAD, but this prognostic value was not existed on the outcome of MI in stable CAD

CAD is one of the leading diseases that cause the morbidity and mortality around the world, which affects the global human population and healthy quality. Our results confirmed that the highest levels of GDF-15 was independently associated with the adverse outcome of all-cause death and cardiovascular death in ACS patients, the association was the sustained in stable CAD patients. We also concluded that the highest concentrations of GDF-15 was the significant warning signal for the adverse outcomes of all-cause death, cardiovascular death and MI in CAD patients. But the highest concentrations of GDF-15 were not as strongly related to the prognosis of MI as all-cause death or cardiovascular death, which may be due to the dysfunction of underlying myocardial damage in MI was not as severe as the degree of death (29, 30). We demonstrated that GDF-15 has performed well in determining the prognosis in ACS and stable angina patients. However, previous meta-analyses have drawn the conclusion only for patients with ACS. After considering patients with coronary artery disease, which included those with stable angina, the present meta-analysis differs itself as a comprehensive appraisal of all available data in CAD patients about the independently prognostic effect of GDF-15.

The condition that GDF-15 as a prognostic biomarker for ACS patients has been recognized, especially in those with NSTEMI (9, 24). However, it is unclear from previous studies whether the prognostic value of GDF-15 can be generalized to the CAD population, that is, whether the prognostic value of GDF-15 is as significant in patients with stable CAD as it is in patients with ACS. Many studies have shown a positive correlation between increased GDF-15 levels and the severity of the disease of CAD itself, for example, one study found that GDF-15 concentration was correlated with cardiac ejection fraction negatively, but with Gensini score, the number of implanted stents and the length of stay-in hospital days, the association was positively. Another study introduced that the proportion of GDF-15 concentrations >1,800 ng/L was markedly higher in ACS patients than those with stable CAD. Our meta-analysis confirmed that the predictive power of the highest levels of GDF-15 for MI was mainly driven by ACS and not stable CAD. The reason why the highest concentrations of GDF-15 has significant prognostic value for ACS patients than stable CAD patients is that the pathological mechanism, inducing factors of ACS and stable CAD are different, although the blood vessels of stable CAD patients was observed stenosis in different degrees, cardiomyocytes do not become severe ischemic and the injury received are relatively mildly (31, 32).

### 4.3. The highest concentrations of GDF-15 has both short-term predictive value and long-term prognosis effect in patients with CAD

The risk prediction and assessment of patients with CAD is a continuous process. GDF-15 was different from other biomarkers which reflected the myocardial necrosis, its properties were relatively stable. As time goes by, plasm circulating GDF-15 levels remains remarkably stable both in the acute setting and the period of stabilization in patients with CAD. Data form the Fragmin and Fast Revascularization During Instability in Coronary Artery Disease-2 trial (FRISC-2) indicated that average GDF-15 concentrations decreased by only 4% during the time of 4–6 months (28), which suggested that GDF-15 primarily reflects the stress state of the body in chronic disease and the burden of it in these patients. This is different from troponin, hs-CRP, and NT-proBNP, which have great dynamic changes in the process of disease occurrence and development (28). The persistent relation between GDF-15 levels and long-term outcomes in CAD patients partly owed to its stability over time. Our meta-analysis confirmed that the highest concentrations of GDF-15 tested on admission can predict all-cause death, cardiovascular death and MI both during the follow up of short-term and long-term among patients with CAD. However, due to the individual differences in GDF-15 plasm levels and the differences in GDF-15 levels at different stages of disease progression, more studies are needed to explore the time points for appropriate monitoring of GDF-15 plasm levels.

The Thrombolysis In Myocardial Infarction (TIMI) score and Global Registry of Acute Coronary Events (GRACE) score are used to assess the risk of adverse cardiovascular events and prognosis in patients with ACS, but based only on clinical characteristics (33–38). GDF-15 is a new indicator that closely related to cardiovascular diseases, which with high sensitivity and specificity. It is up-regulated in a variety of physiological tissues, such as: placental, prostate, liver, kidney (23), increased levels of GDF-15 have also been discovered in the aspects of myocardial damage such as myocardial infarction, heart failure and myocardial lesions (39). GDF-15 can increase in the early stage and reach the peak in a short time, so it probably should be used as a supplement to other myocardial injury biomarkers, which is helpful for the early risk stratification and guideline prognosis of CAD in future clinical practice.

At present, continuously measurement of hs-troponin is the recommended advice to assess the prognosis among patients with ACS in the guidelines (40). With the development of technical measurement and sensitivity of detection, GDF-15 at admission is expected to improve the overall predictive ability of traditional cardiovascular risk factors in CAD patients. Next, detailed studies of GDF-15 should pay attention to profound understanding of its physiopathologic features and mechanism role in myocardial injury, which can define the clinical role of this novel biomarker better and reveal new treatment targets about GDF-15.

## 5. Conclusion

Among the highest GDF-15 levels on admission in CAD patients, there was a risk of all-cause death, cardiovascular death, MI and stroke after adjusting clinical characteristics. The relationship was persisted in the outcome of all-cause death, cardiovascular death and MI after adjustment for hs-troponin T, cystatin C, hs-CRP and NT-proBNP, but was unclear in stroke. The highest concentrations of GDF-15 had a lower predictive effect on MI than all-cause death and cardiovascular death in CAD patients. The highest levels of GDF-15 on admission can provide independent risk information for individual cardiovascular events in patients with ACS and stable CAD, which may help clinicians to identify the high-risk patients and adopt positive interventions.

## 6. Limitations

Our meta-analysis and systematic review had several limitations. First, there was no agreed standard definition in cut-off values for GDF-15, our analysis was performed according to <1,200 ng/L, 1,200–1,800 ng/L, and >1,800 ng/L and the quartile of GDF-15 levels. Although our results drawn the conclusion that high GDF-15 values predict the adverse individual cardiovascular events independently, we cannot define the optimized cut-off value of GDF-15 for predicting prognoses. Second, the types of study design included clinical trials and cohort studies. The cohort studies are observational studies, which contained unselected patients. They are more heterogeneous and may have greater potential for variation in underlying prognosis. Third, the different treatment strategies that CAD patients adopted before collecting blood samples might influence the GDF-15 concentrations.

## Data availability statement

The original contributions presented in the study are included in the article/Supplementary material, further inquiries can be directed to the corresponding authors.

## Author contributions

SZ contributed to the acquisition, analysis, and interpretation of data. PH resolved the disagreement during data extraction. JL, QZ, and XY contributed to the drawing of the forest plots. JW and YC gave specific guidance. All authors contributed to the article and approved the submitted version.

## Funding

This study was supported by the National Key R&D Program of China (2020YFC1512700, 2020YFC1512705, 2020YFC1512703), National Natural Science Foundation of China (81873953, 82172178), National S&T Fundamental Resources Investigation Project (2018FY100600, 2018FY100602), Key R&D Program of Shandong Province (2021ZLGX02, 2021SFGC0503), Taishan Pandeng Scholar Program of Shandong Province (tspd20181220), Taishan Young Scholar Program of Shandong Province (tsqn20161065, tsqn201812129), Youth Top-Talent Project of National Ten Thousand Talents Plan and Qilu Young Scholar Program.

## Conflict of interest

The authors declare that the research was conducted in the absence of any commercial or financial relationships that could be construed as a potential conflict of interest.

## Publisher’s note

All claims expressed in this article are solely those of the authors and do not necessarily represent those of their affiliated organizations, or those of the publisher, the editors and the reviewers. Any product that may be evaluated in this article, or claim that may be made by its manufacturer, is not guaranteed or endorsed by the publisher.

## Supplementary material

Supplemental FIGURE 1 Funnel plot analysis of publication bias about all-cause death, cardiovascular death, MI, stroke for studies comparing the highest and lowest concentrations of GDF-15.

Supplemental FIGURE 2 Forest plot showing the HR and 95% CI of all-cause death for different disease types in patients comparing the highest and lowest concentrations of GDF-15.

Supplemental FIGURE 3 Forest plot showing the HR and 95% CI of cardiovascular death for different disease types in patients comparing the highest and lowest concentrations of GDF-15.

Supplemental FIGURE 4 Forest plot showing the HR and 95% CI of MI for different disease types in patients comparing the highest and lowest concentrations of GDF-15.

Supplemental FIGURE 5 Forest plot showing the HR and 95% CI of all-cause death for different follow-up time in patients comparing the highest and lowest concentrations of GDF-15.

Supplemental FIGURE 6 Forest plot showing the HR and 95% CI of cardiovascular death for different follow-up time in patients comparing the highest and lowest concentrations of GDF-15.

Supplemental FIGURE 7 Forest plot showing the HR and 95% CI of MI for different follow-up time in patients comparing the highest and lowest concentrations of GDF-15.
