# Supplementary figures and images for: Prognostic value of growth differentiation factor-15 in patients with coronary artery disease: A meta-analysis and systematic review
Source: Front Cardiovasc Med. 2023 Feb 10;10:1054187. doi: 10.3389/fcvm.2023.1054187 (PMC9950748; doi:10.3389/fcvm.2023.1054187)

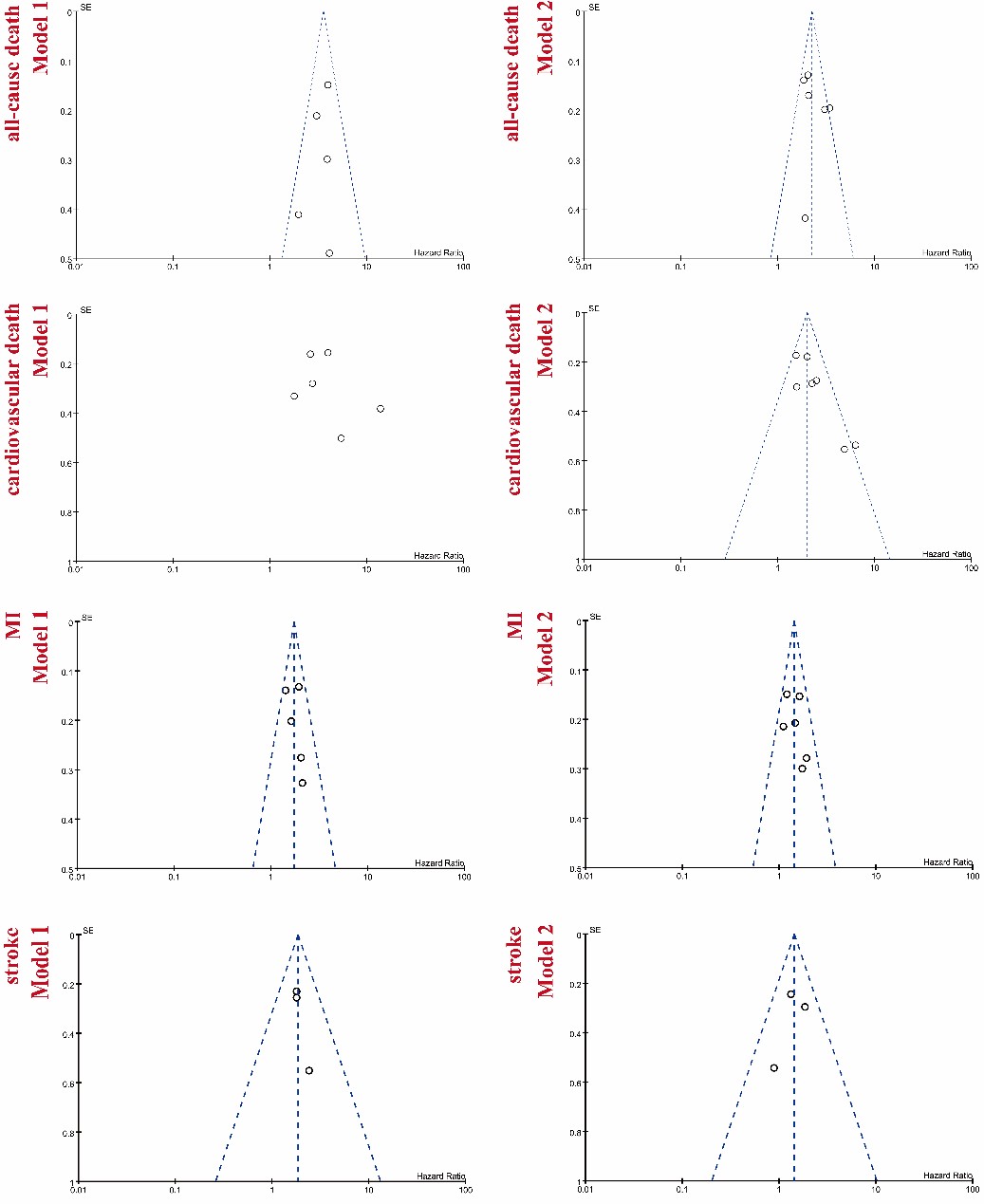

Supplement: Supplemental FIGURE 1 — Funnel plot analysis of publication bias about all-cause death, cardiovascular death, MI, stroke for studies comparing the highest and lowest concentrations of GDF-15. [file Image_1.JPEG]

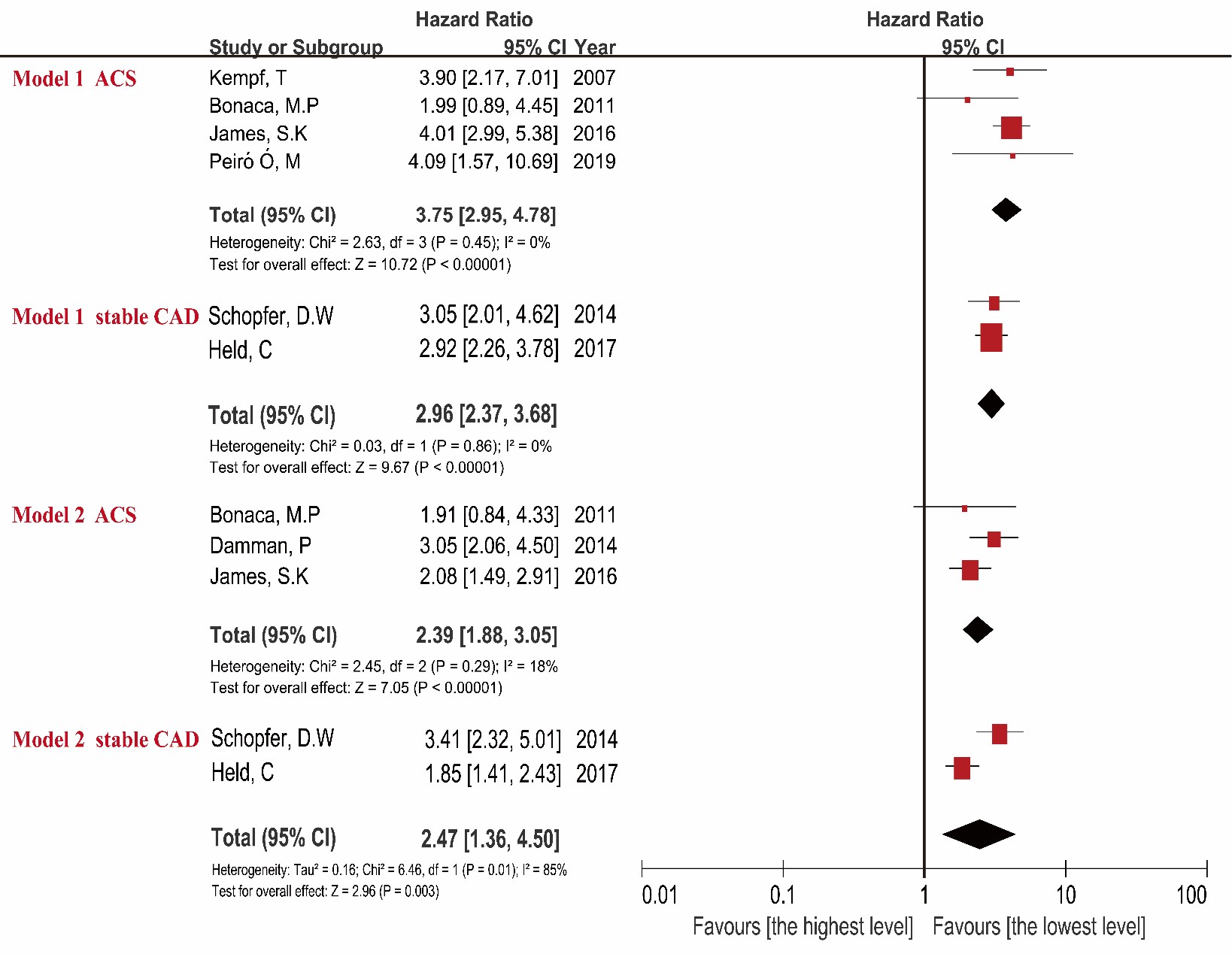

Supplement: Supplemental FIGURE 2 — Forest plot showing the HR and 95% CI of all-cause death for different disease types in patients comparing the highest and lowest concentrations of GDF-15. [file Image_2.JPEG]

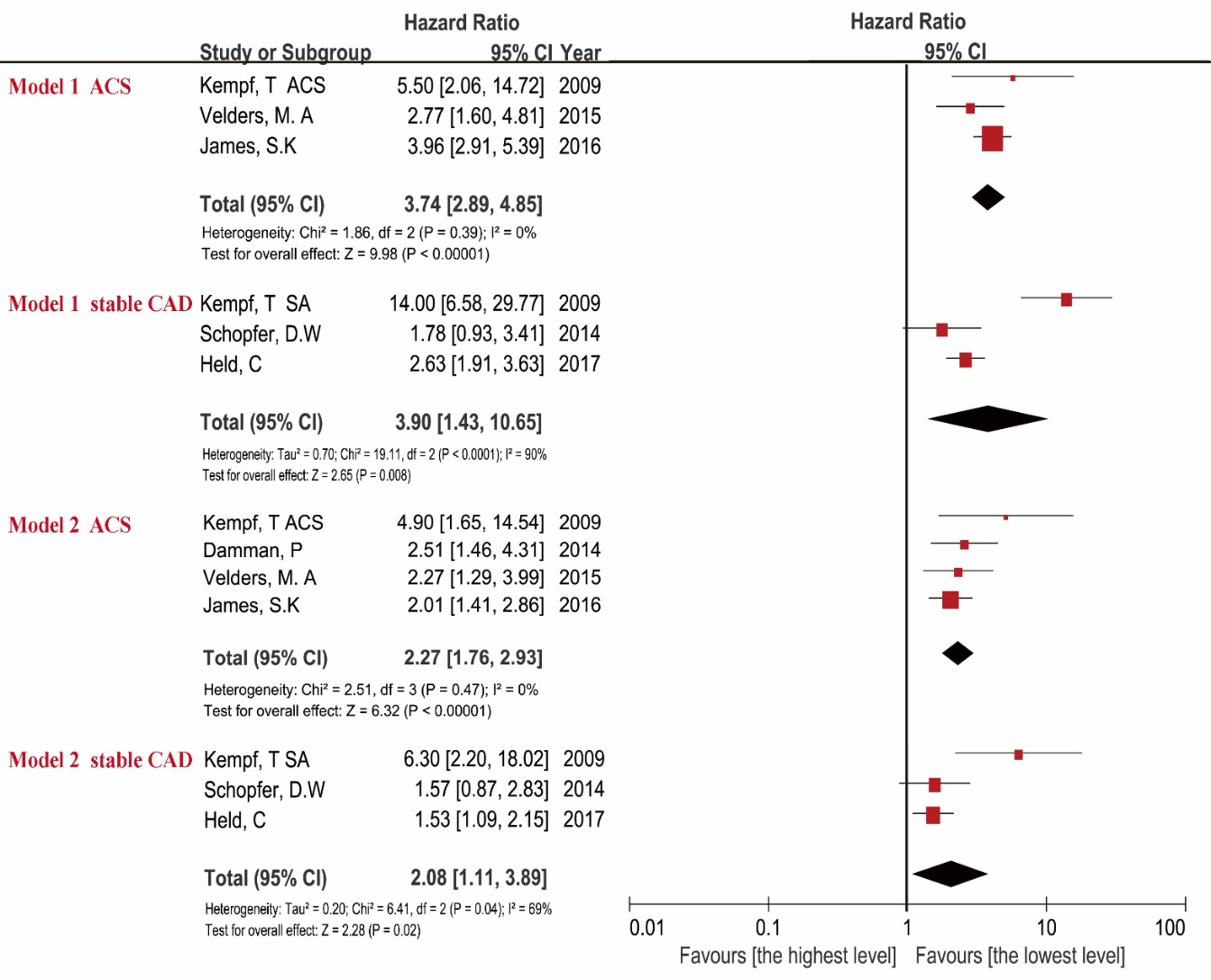

Supplement: Supplemental FIGURE 3 — Forest plot showing the HR and 95% CI of cardiovascular death for different disease types in patients comparing the highest and lowest concentrations of GDF-15. [file Image_3.JPEG]

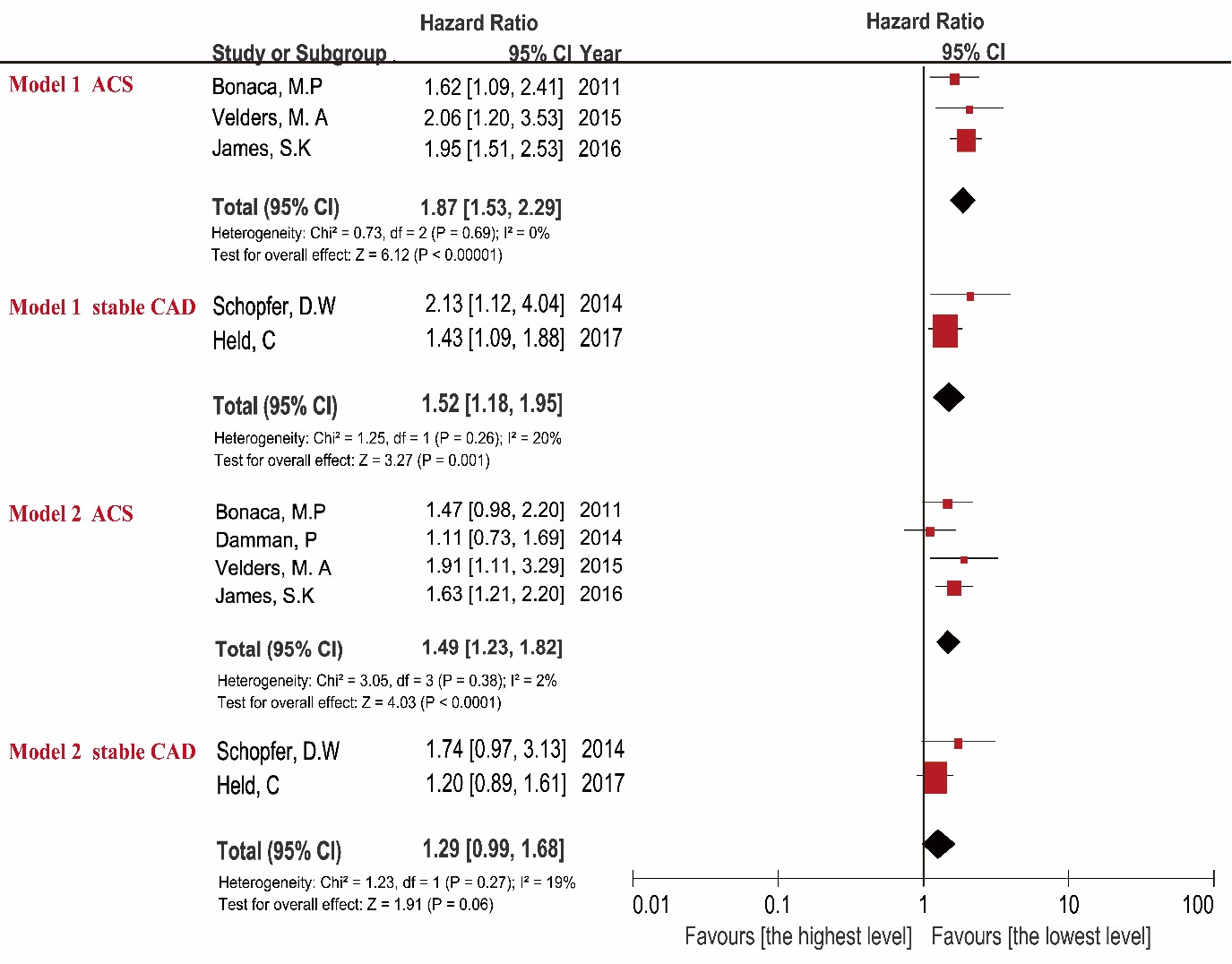

Supplement: Supplemental FIGURE 4 — Forest plot showing the HR and 95% CI of MI for different disease types in patients comparing the highest and lowest concentrations of GDF-15. [file Image_4.JPEG]

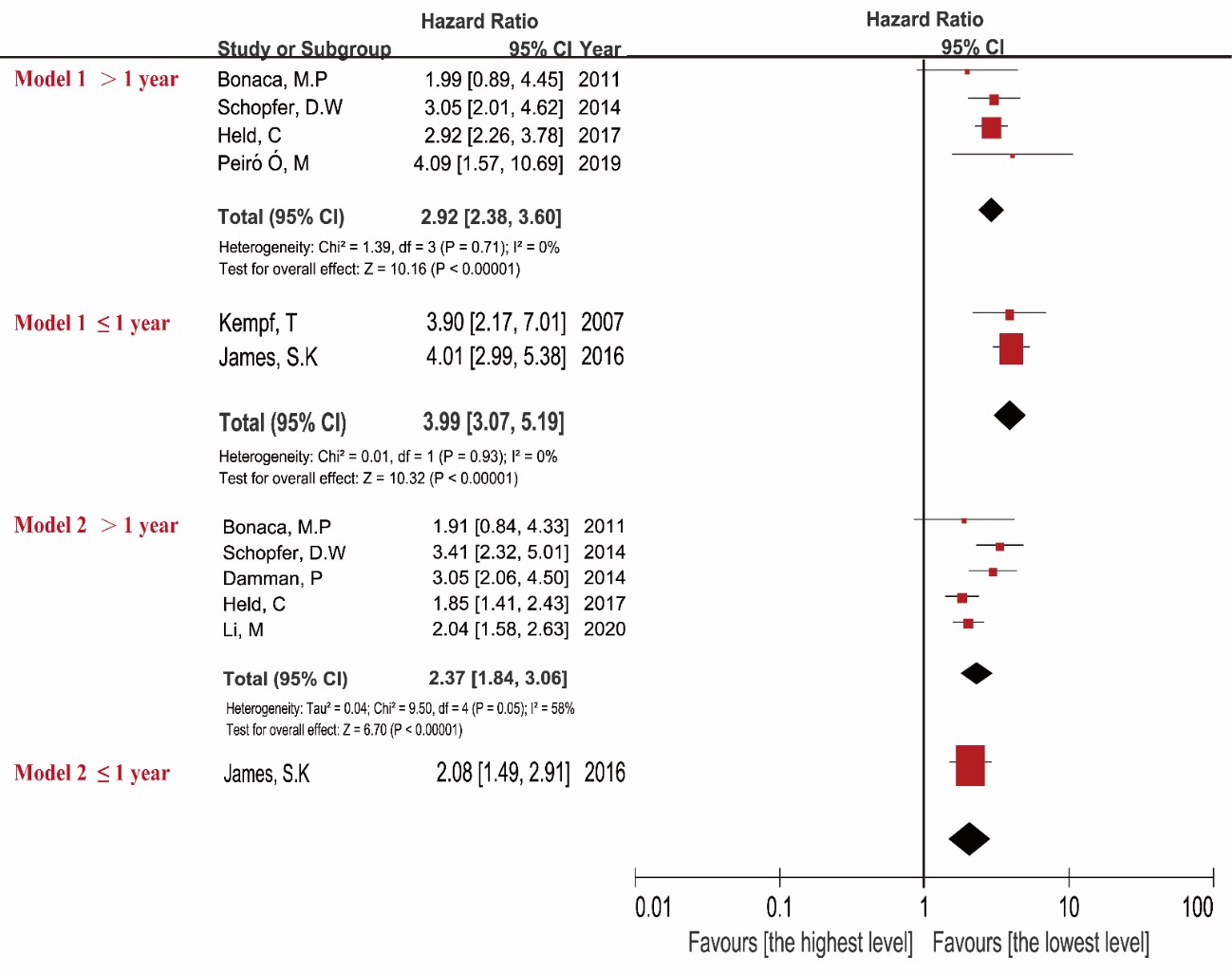

Supplement: Supplemental FIGURE 5 — Forest plot showing the HR and 95% CI of all-cause death for different follow-up time in patients comparing the highest and lowest concentrations of GDF-15. [file Image_5.JPEG]

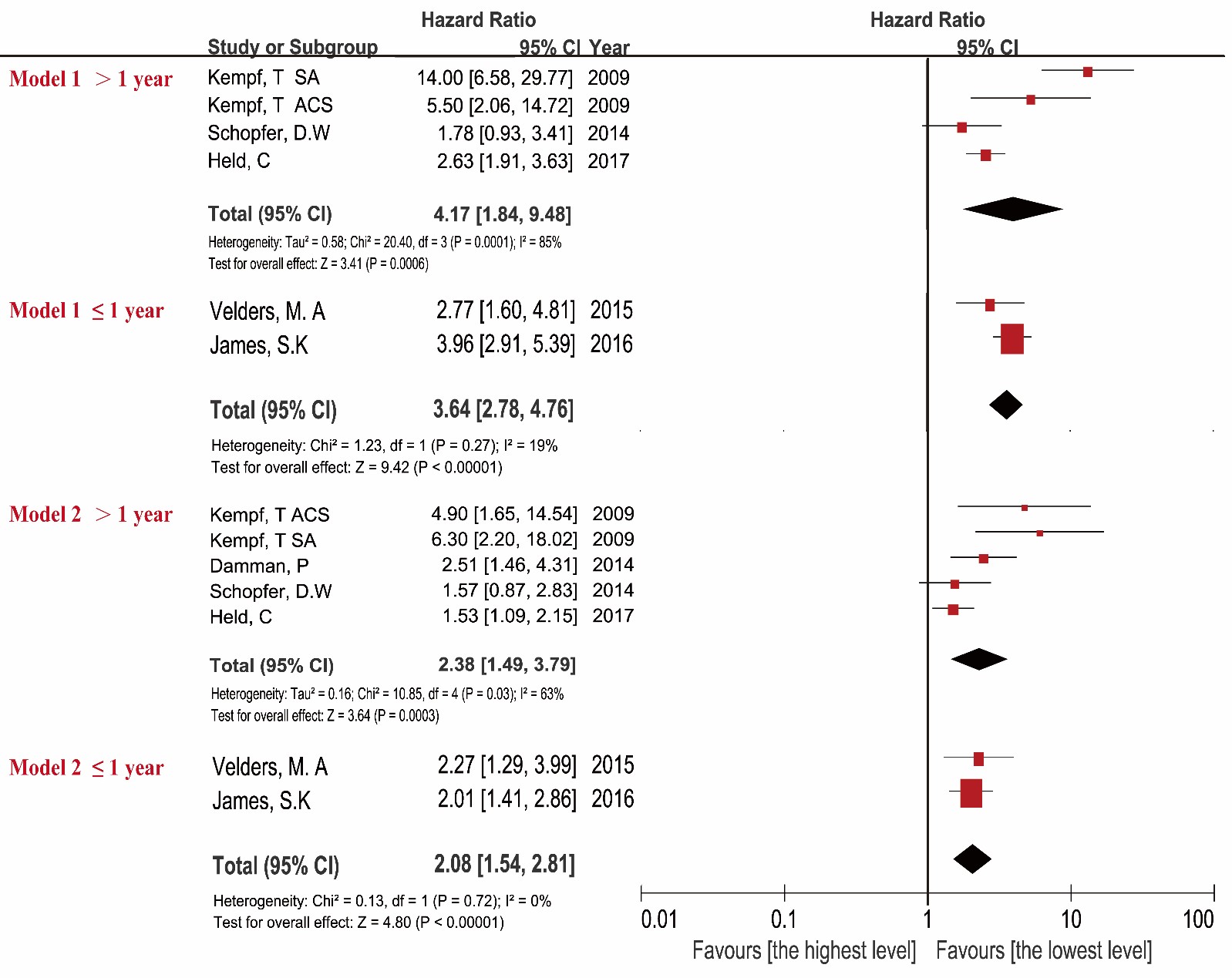

Supplement: Supplemental FIGURE 6 — Forest plot showing the HR and 95% CI of cardiovascular death for different follow-up time in patients comparing the highest and lowest concentrations of GDF-15. [file Image_6.JPEG]

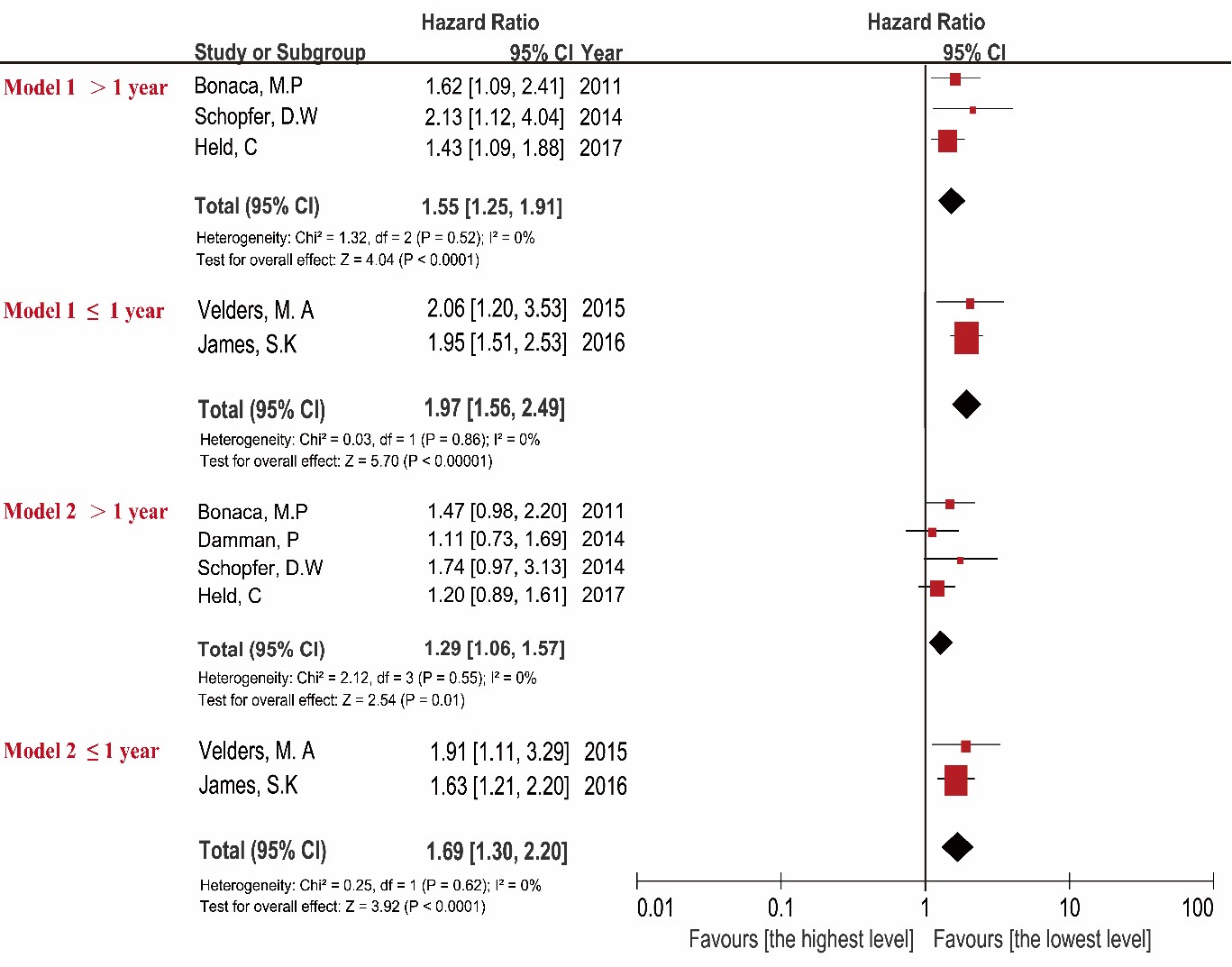

Supplement: Supplemental FIGURE 7 — Forest plot showing the HR and 95% CI of MI for different follow-up time in patients comparing the highest and lowest concentrations of GDF-15. [file Image_7.JPEG]
