# Supplementary material for: Prognostic value of growth differentiation factor-15 in patients with coronary artery disease: A meta-analysis and systematic review
Source: Front Cardiovasc Med. 2023 Feb 10;10:1054187. doi: 10.3389/fcvm.2023.1054187 (PMC9950748; doi:10.3389/fcvm.2023.1054187)
Supplement: Supplementary file 8 [file Table_1.DOCX]

Supplemental Table 1. The quality of studies with NOS scores

| **Studies** | **Selection** | **Comparability** | | **Exposure** | **Stars** |
| --- | --- | --- | --- | --- | --- |
| Peiró ÓM, et al 2019 | 3 | 1 | 2 | | 6 |
| Lindholm D, et al 2017 | 2 | 2 | 2 | | 6 |
| Held C, et al 2017 | 2 | 1 | 3 | | 6 |
| James SK, et al 2016 | 3 | 1 | 3 | | 7 |
| Damman P, et al 2014 | 3 | 2 | 3 | | 8 |
| Kempf T, et al 2009 | 3 | 1 | 2 | | 6 |
| Velders MA, et al 2015 | 3 | 2 | 3 | | 8 |
| Li M, et al 2020 | 3 | 1 | 2 | | 6 |
| Kempf T, et al 2007 | 2 | 2 | 3 | | 7 |
| Bonaca MP, et al 2011 | 3 | 2 | 3 | | 8 |

NOS = newcastle-ottawa scale.
