# Supplementary material for: Prognostic value of growth differentiation factor-15 in patients with coronary artery disease: A meta-analysis and systematic review
Source: Front Cardiovasc Med. 2023 Feb 10;10:1054187. doi: 10.3389/fcvm.2023.1054187 (PMC9950748; doi:10.3389/fcvm.2023.1054187)
Supplement: Supplementary file 9 [file Table_2.docx]

**Supplemental Table 2.** The HR and 95% CI of individual cardiovascular events at middle concentrations of GDF-15

| Individual cardiovascular events | Model | No. of studies | HR [95%CI] | p-value | I^2^(%) | p-value^b^ |
| --- | --- | --- | --- | --- | --- | --- |
| all-cause death | model 1 | 4 | 2.18[1.56,3.04] | ＜0.0001 | 82 | 0.0008 |
|  | model 2 | 3 | 1.38[1.13,1.67] | 0.001 | 0 | 0.68 |
| cardiovascular death | model 1 | 5 | 1.84[1.51,2.24] | ＜0.0001 | 0 | 0.78 |
|  | model 2 | 4 | 1.40[1.06,1.86] | 0.02 | 0 | 0.80 |
| MI | model 1 | 4 | 1.33[1.05,1.69] | 0.02 | 51 | 0.11 |
|  | model 2 | 4 | 1.23[0.96,1.58] | 0.11 | 54 | 0.09 |
| stroke | model 1 | 2 | 1.80[1.31,2.46] | 0.0002 | 0 | 0.79 |
|  | model 2 | 2 | 1.65[1.18,2.31] | 0.004 | 0 | 0.56 |

Model l: age, gender, previous MI, previous percutaneous coronary intervention (PCI), previous coronary artery bypass grafting (CABG), body mass index (BMI), diabetes mellitus, hypertension, admission heart rate, history of heart failure, peripheral arterial disease, smoking.

Model 2: included model 1, with the addition of hs-troponin T, N-terminal pro B-type natriuretic peptide (NT-proBNP), cystatin C, and high-sensitivity C-reactive protein (hs-CRP).

HR: hazard ratio.
